# Further evidence for the interaction of central and peripheral processes: the impact of double letters in writing English words

**DOI:** 10.3389/fpsyg.2013.00729

**Published:** 2013-10-10

**Authors:** Sonia Kandel, Ronald Peereman, Anna Ghimenton

**Affiliations:** ^1^Laboratoire de Psychologie et NeuroCognition, Centre National de la Recherche Scientifique, UMR 5105, University of Grenoble AlpesGrenoble, France; ^2^Institut Universitaire de FranceParis, France; ^3^Département of Parole and Cognition, Université Grenoble Alpes, GIPSA-LAB CNRS UMR 5216Grenoble, France; ^4^Institut de Linguistique et Phonétique Générales et Appliquées (ILPGA), Université Paris 3 – Sorbonne NouvelleParis, France

**Keywords:** double letters, handwriting, cascaded processing, spelling, central processing, peripheral processing

## Abstract

Most studies on spelling processes suppose that the activation of orthographic representations is over before we start to write. The goal of the present study was to provide evidence indicating that the orthographic representations activated during spelling production interact continuously with the motor processes during movement production. We manipulated gemination to assess the influence of the orthographic properties of words on the kinematic parameters of production. Native English-speaking participants wrote words containing double letters and control words on a digitizer [e.g., DI*SS*IPATE (Geminate) and DI*SG*RACE (Control)]. The word pairs shared the initial letters and differed on the presence of a doublet at the same position. The results revealed that latencies were shorter for Geminates than Controls, indicating that spelling processes were facilitated by the presence of a doublet in the word. Critically, the impact of letter doubling was also observed during production, with shorter letter durations (e.g., D, I, S) and intervals (DI, IS) for Geminates than Controls. Letter doubling therefore affected the whole process of word writing: from spelling recall to movement preparation and production. The spelling processes that were involved before movement initiation cascaded into processes that regulate movement execution. The activation spread onto peripheral processing until the production of the doublet was completely programmed (e.g., letter S).

## Introduction

When we have to write a word, we activate its orthographic representation so we can get information on its letter components. Most studies on spelling processes suppose that the activation process is over before we start to write (Bonin et al., 2001; Zhang and Damian, 2010; Afonso and Álvarez, 2011; Qu et al., 2011). These central processes are followed by peripheral processes that activate motor programs containing information on letter shape, stroke order, and direction (Teulings et al., 1983; Van Galen et al., 1989; Van Galen, 1991). The timing of motor production for a given letter depends on its shape, of course, but may also be constrained by spelling processes. Recent data indicate that the way orthographic representations code letters has an impact on handwriting production (Roux et al., 2013). In other words, there is an interaction between central and peripheral processes. Spelling processes are still active during movement production. The spelling processes cascade on to the motor processes. The present study investigated whether particular orthographic properties of words such as double letters (e.g., DI*SS*IPATE), will affect letter production. The on-line kinematic measures on the handwriting movement provided by a digitizer allowed us to analyse the effect of letter doubling before the participants started to write the word (central processes) and while they wrote it (peripheral processes). In this way, we could examine the locus of the interaction. We observed *how* and *when* the processing of words containing doublets affected the writing movements.

The idea of cascading processing is not new and has been extensively discussed in spoken word production (see for example, Kawamoto et al., 1998; Rastle et al., 2000). Writing research on this issue is, in contrast, rather scarce. One study examined the effects of word frequency and orthographic regularity^1^ with a spelling to dictation task (Delattre et al., 2006). Central processes were measured by latency and peripheral processes by writing duration. The results revealed frequency and orthographic regularity effects on latencies. Regarding peripheral processing, words with irregular spellings yielded longer durations than words with regular spellings. Word frequency did not yield a significant effect. The authors concluded that handwriting movements are affected by central orthographic processes after movement initiation. They suggest that spelling processes cascade onto motor processes.

Another recent study examined the cascading process with a more fine-grained methodology (Roux et al., 2013). Instead of measuring the duration of the whole word (Delattre et al., 2006), peripheral processing was examined by measuring the duration of each letter in the word. The latency values, together with the durations of each letter, provide insight into the locus of the cascade throughout the word. The participants copied orthographically irregular French words (e.g., FEMME, woman), regular words (e.g., FORME, shape) and pseudo-words (e.g., FARNE) on a digitizer. The results indicated that sublexical and lexical processing produced different kinds of cascades. The effect of lexicality (regular words vs. pseudo-words) was observed on the duration of the initial letters of the items. For orthographic regularity (regular words vs. irregular words), the extent of the cascade depended on the position of the irregularity. When the irregularity was in word initial, its impact was only observed on the first letter. For the words presenting the irregularity at the end, letter durations were systematically longer for irregular than regular words throughout the whole word. In other words, the spelling processes were active from letter position 1 until the irregular portion of the word was written. Although these results provide evidence for an interaction between central and peripheral processes, the critical influence of the position of the irregular grapheme was observed in *post-hoc* analyses. The words involved in theses analyses might differ on other dimensions that the authors did not control for. The present study assessed the impact of high-order variables on the dynamics of word production with another spelling specificity, namely letter doubling. The presence of double letters is an interesting orthographic characteristic of words that has not yet been investigated during on-line word production. Orthographic regularity effects result from conflicting alternative spellings generated by lexical and sub-lexical processes (Rapp et al., 2002). Gemination instead, is supposed to be specifically coded in the orthographic representation of words.

Most data on doublet representation in words comes from case studies of dysgraphic patients. McCloskey et al. (1994) presented the case of an English-speaking patient HE who produced much more errors when writing words containing doublets than control words. For 83% of the errors in the words containing doublets he doubled the wrong letter (e.g., LOOK written LOKK). Tainturier and Caramazza's (1996) dysgraphic English-speaking patient behaved in a similar way. His writing also revealed that doublets produce different error patterns than letters that appear twice within a word but not in adjacent positions (e.g., *C*A*C*TUS) or as letter chunks that represent a phoneme (e.g., RO*CK*ET where CK = /k/). Other studies conducted in Italian also present cases supporting the idea that orthographic representations of words containing doublets have a specific coding (Venneri et al., 1994; Miceli et al., 1995).

Studies on spelling acquisition in English and French also provide evidence for a specific processing of double letters. In an experiment by Cassar and Treiman (1997) English-speaking 1st graders considered pseudo-words that had an embedded “legal” and frequent doublet (e.g., LL) as more word-like than pseudo-words that had an “illegal” doublet (e.g., HH). Further research indicated that very early in the acquisition process the children are sensitive to the position of the doublet within the word as well. For example, Pacton and colleagues (Pacton et al., 2001; Danjon and Pacton, 2009) presented data in which French-speaking 1st to 4th graders preferred pseudo-words that had the doublet in medial position like FO*MM*IR than pseudo-words with a doublet in initial position (e.g., *FF*OMIR), which is illegal in French. Interestingly, 6-year old children learning pseudo-words containing initial doublets (RREK) produce transpositions of the doublet feature to the final position when spelling the words (REKK; Wright and Ehri, 2007). Convergent evidence was reported by Fayol et al. (2010) in French university students. Pseudo-words that contained an infrequent doublet (e.g., DD in TI*DD*UNAR) led to transpositions of the doublet feature to a consonant that is more frequently doubled in French (such as NN in TIDU*NN*AR). Conversely, transposition errors were less numerous when the spelling of the pseudo-words contained a frequent doublet in French. These findings are in line with the neuropsychological data and suggest a specific coding for doublets: letter identity and quantity seem to be represented separately. These observations also rely on off-line measures and do not provide information on *how* and *when* the doublet feature influences the motor process.

A few typing studies presented on-line data on doublet processing but they did not investigate the interaction between central and peripheral processes. Their stimuli were non-sense consonant letter strings and not words. Sternberg et al. (1983; also Sternberg et al., 1990) measured the duration of inter-key intervals that either contained double letters or not. The results revealed that the duration was a linear function of the number of elements in the sequence (e.g., SFCRZ > SFCR). For the sequences of equal length but containing double letters the durations were shorter than for the ones not containing double letters (e.g., SFCRZ > SCCRZ). They were equivalent to the durations of the sequences that contained four letters (e.g., SCCRZ = SFCR). The authors accounted for these results in terms of motor production and considered that “the production of strings that include a doublet indicates that the two strokes of the doublet are contained in the same action unit” (Sternberg et al., 1990, p. 41). However, the authors were not concerned by orthographic representations and did not argue in favor of a specific level for double letter coding.

The neuropsychological studies, together with the data on spelling acquisition and typing suggest that double letters could be coded at a different level of orthographic representation. This level could be different from other frequent two letter clusters as complex graphemes. The typing results and the outcome of the neuropsychological observations were integrated by Glasspool and Houghton (2005) in a computational spelling model that includes a specific “geminate” node in its architecture. However, the question on how the gemination feature affects peripheral processing is still open. The goal of the present study was to provide on-line data on *how* and *when* this kind of orthographic coding affects movement production.

Several studies revealed that the structural characteristics of words can affect the movement dynamics of typewriting (Weingarten et al., 2004; Weingarten, 2005). Data on handwriting also provide evidence that specific letter clusters affect movement production (Kandel et al., 2006, 2011). For example, in French, letter A is pronounced /a/ like in the word CL*A*VIER (keyboard) and will be processed as a single unit. But when A is associated to I, like in PR*A*IRIE (meadow), it is pronounced /ε/ because it belongs to the complex grapheme AI. The timing of motor production for writing letter A in CL*A*VIER is shorter than when producing it in PR*A*IRIE (Kandel and Spinelli, 2010). The writing system processes AI as a cluster and modulates the way A will be produced. Processing complex graphemes also affects the timing of the preceding letter. The duration of L in CL*A*VIER was shorter than the duration of R in PR*A*IRIE^2^. So the way orthographic representations code phonology affects movement production.

In the present study we conducted an experiment where the participants wrote English words that contained double letters (e.g., DI*SS*IPATE, Geminate words hereafter). In English, letter doubling does not affect pronunciation. We compared their production to words that shared the initial letters but had no embedded double letters (e.g., DI*SG*RACE, Control words hereafter). We measured latency (i.e., the time before movement initiation) because it gives insight into central processing. Regarding peripheral processing, we measured letter duration (e.g., D, I, S and S or G, respectively) and the duration of the intervals between letters (e.g., between D and I, I and S, S and S or S and G, respectively). If the orthographic representations the system activates when we have to write a word code double letters as whole units, the information on letter doubling should be processed before movement initiation. It follows that if central and peripheral processing interact, we should observe the central processing cascade and remain active during the production of the initial letters of the word. This implies that there should be latency, letter duration and interval duration differences between Geminate and Control words. Thus, DIS should exhibit different durational patterns in Geminate (DI*SS*IPATE) and Control (DI*SG*RACE) words. At the more local level—i.e., when the doublet occurs—we predict that the fourth letter (the second S in DISSIPATE and G in DISGRACE) should always be longer in Control than Geminate words. Indeed, if the orthographic representation codes double letters, the second S in DISSIPATE should be anticipated and thus programmed beforehand. When the S is being produced, the system should only process the local parameters required for letter production. In contrast, the programming of G in DISGRACE should not benefit from a specific anticipation for doubling, so its production should be more time consuming than the S of DISSIPATE.

## Method

### Participants

There were 20 native English-speaking participants that were attending Harvard University for summer courses. The experimental design was approved by the Harvard IRB committee. All the participants were right-handed, had normal or corrected-to-normal vision, and no motor or hearing disorders. Their ages ranged from 20 to 30 years old. They were unaware of the purpose of the experiment. They gave written consent for their participation in the experiment and participated on a voluntary basis.

### Materials

We selected 28 words (see Appendix). Fourteen words had a doublet at positions 3 and 4 (DI*SS*IPATE). We matched these Geminate words to words that shared the same three initial letters but had no doublet (DI*SG*RACE). The words in the two conditions were matched for word frequency, number of letters and syllables, orthographic similarity with other words (orthographic neighborhood), and bigram frequency (Table 1). On average, bigram frequency at the position of the doublet (SS, SG) was lower than for the first (DI) and the second (IS) bigrams of geminate and control words (by-type values: 2905 and 2655 for the first and the second bigrams, respectively; corresponding by-token values: 8545 and 12,980).

**Table 1:** **Characteristics of the words used in the experiment**.

**Variables**	**Geminates**	**Controls**	***p*-values (*t*-test)**
Word frequency (pm)^a^	26.17	14.18	ns
Length (letters)	7.14	6.93	ns
Lexical neighborhood^a^^,^^b^	2.22	2.14	ns
Bigram frequency at the doublet position^a^^,^^c^	1539 (4266)	1359 (4846)	ns (ns)

a Based of the Celex database (Baayen et al., 1995).

b As determined by the Levenshtein distance metric (Yarkoni et al., 2008).

c Bigram frequency computed on the basis of Celex, per type (and per token).

### Procedure

The experiment was conducted with *Ductus* (Guinet and Kandel, 2010). At the beginning of each trial, the participants heard an auditory signal and saw a fixation point at the center of a laptop screen. This fixation point was replaced by a word written in upper-case Times New Roman size 18. The participants were instructed to write the word they saw as soon as it appeared on the screen. They had to write it at a normal speed. They wrote the word with a special pen (Intuos Inking Pen) on a lined paper (vertical limit = 8 mm, horizontal limit = 17 cm) that was stuck to a digitizer (Wacom Intuos 2, sampling frequency 200 Hz, accuracy 0.02 mm). They had to write the words in upper-case letters and lift the pen between letters in a small upward-downward wrist movement. When the participant finished writing a word, the experimenter clicked on a button to present the following word. Prior to the experiment, the participants practiced lifting the pen between letters by writing their names several times, until they thought they could do it “spontaneously” for the purposes of the experiment.

We presented the 28 words in two blocks of 14 stimuli. The words were randomized across participants. There were 10 filler items so that there were more words that did not have double letters than words with double letters. There were two practice items before the beginning of the experimental session. The participants were tested individually in a quiet room. The whole session lasted 10–15 min.

### Data processing and analysis

To obtain the measures on latencies, letter and inter-letter interval durations, we used the data analysis module provided by *Ductus* (Guinet and Kandel, 2010). The data were smoothed with a Finite Impulse Response filter (Rabiner and Gold, 1975) with a 12 Hz cut-off frequency. Letter duration referred to the time the participants took to write a letter. To investigate whether gemination processing cascades throughout the initial letters of the word, we had to compare the durations of letters that are made up of a different number of strokes (e.g., in DI*SS*IPATE/DI*SG*RACE, D has 3 strokes, I has 1, S has 3, and G has 4). To control for this point, we normalized the duration values with respect to the number of strokes per letter. The letter segmentation was determined on the basis of a previous up-stroke/down-stroke analysis of each upper-case letter of the alphabet (cf. Kandel and Spinelli, 2010; Spinelli et al., 2012). We also measured the duration of the intervals between letters. For example, in DI*SS*IPATE/DI*SG*RACE, we measured the time that the pen was in the air at the intervals between D and I, I and S, S and S or S and G, respectively. The interval duration was defined as the time period in which two letters were separated by a pen lift. The letter end corresponded to pressure = 0 and the onset of the following letter corresponded to pressure > 0. Finally, latency concerned the time between the presentation of the word on the screen and the moment at which the participant started to write it (pressure > 0).

## Results

We conducted ANOVAs with word type (geminated, control words) as main within-participants factor, both by participants (*F*1) and by items (*F*2). For the analysis of stroke duration we included letter position as within-participants factor (e.g., for DI*SS*IPATE/DI*SG*RACE): Letter 1 (L1) = D, Letter 2 (L2) = I, Letter 3 (L3) = S, Letter 4 (L4) = S and G. For the analysis of interval durations we included interval position as within-participants factor [e.g., Interval 1 (I1) = DI, Interval 2 (I2) = IS, Interval 3 (I3) = SS and SG].

### Latency

Latencies higher than 3000 ms or below 300 ms were excluded (0.8% of the data). The remaining latencies and letter stroke durations that exceeded 2 standard deviations above or below each participant and item mean were also discarded (1.3% of the data). Mean latencies for geminated words were 1139 ms (*SD* = 276 ms) and 1220 (*SD* = 300 ms) for controls. The analysis indicated that movement initiation was shorter for geminated words than controls, *F*1_(1, 19)_ = 11.86, *p* < 0.01; *F*2_(1, 13)_ = 22.45, *p* < 0.001.

### Letter stroke duration

**Figure 1:**
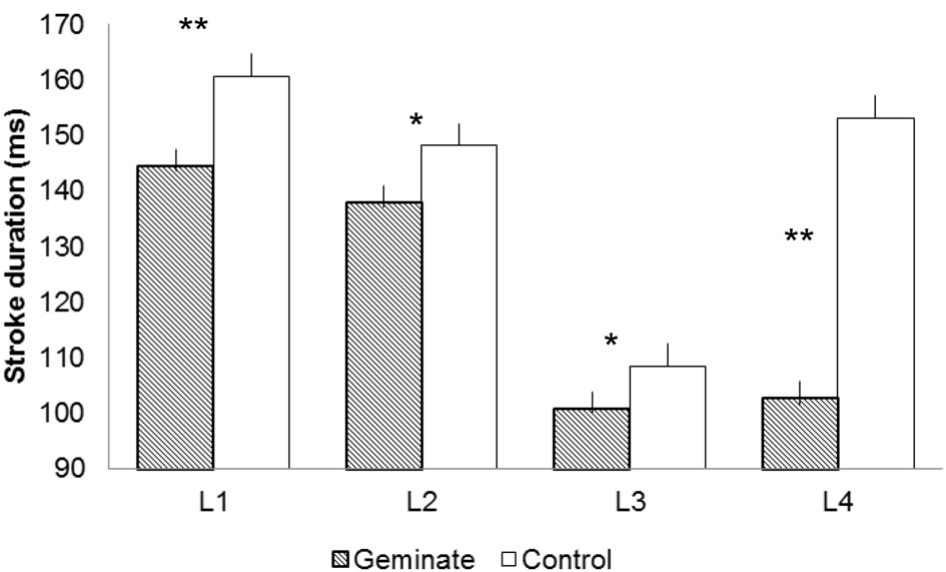
**Letter stroke durations for letters 1–4 [e.g., DISSIPATE/DISGRACE: Letter 1 (L1) = D, Letter 2 (L2) = I, Letter 3 (L3) = S, Letter 4 (L4) = S/G]**. The ^*^ indicates a significant duration difference between Geminate and Control words at a specific location of the word (*F*1 only); ^**^ indicates that the difference is significant for *F*1 and *F*2.

Figure 1 presents the mean letter stroke durations for letters 1–4 for Geminate and Control words. Geminate words yielded shorter stroke durations than controls, *F*1_(1, 19)_ = 286.22, *p* < 0.001; *F*2_(1, 13)_ = 15.03, *p* < 0.001. Letter position yielded a significant effect, *F*1_(3, 57)_ = 236.76, *p* < 0.001; *F*2_(3, 39)_ = 7.75, *p* < 0.001. The interaction between the two factors was significant, *F*1_(3, 57)_ = 159.88, *p* < 0.001; *F*2_(3, 39)_ = 12.34, *p* < 0.001.

Pairwise comparisons revealed that the stroke durations for geminate words were shorter than controls at all positions: for L1, *F*1_(1, 19)_ = 76.37, *p* < 0.001; *F*2_(1, 13)_ = 6.07, *p* < 0.05; for L2, *F*1_(1, 19)_ = 99.90, *p* < 0.001; *F*2_(1, 13)_ < 1; for L3, *F*1_(1, 19)_ = 26.74, *p* < 0.001; *F*2_(1, 13)_ < 1; and for L4, *F*1_(1, 19)_ = 363.74, *p* < 0.001; *F*2_(1, 13)_ = 13.49, *p* < 0.01. The durations remained stable from L3 to L4 (S) in geminates, *F*1_(1, 19)_ = 2.46, ns; *F*2 < 1. In contrast, the durations increased significantly from L3 (S) to L4 (G) in controls, *F*1_(1, 19)_ = 378.98, *p* < 0.001; *F*2_(1, 13)_ = 14.74, *p* < 0.01 (Bonferroni corrected). Latency values correlated positively and significantly with stroke duration at each letter position for geminates: for L1, *R* = 0.47, *p* < 0.05; for L2, *R* = 0.49, *p* < 0.05; for L3, *R* = 0.56, *p* < 0.01; and for L4, *R* = 0.62, *p* < 0.01. The same pattern was observed for controls: for L1, *R* = 0.55, *p* < 0.01; for L2, *R* = 0.45, *p* < 0.05; for L3, *R* = 0.55, *p* < 0.01; and for L4, *R* = 0.64, *p* < 0.01.

### Inter-letter interval duration

**Figure 2:**
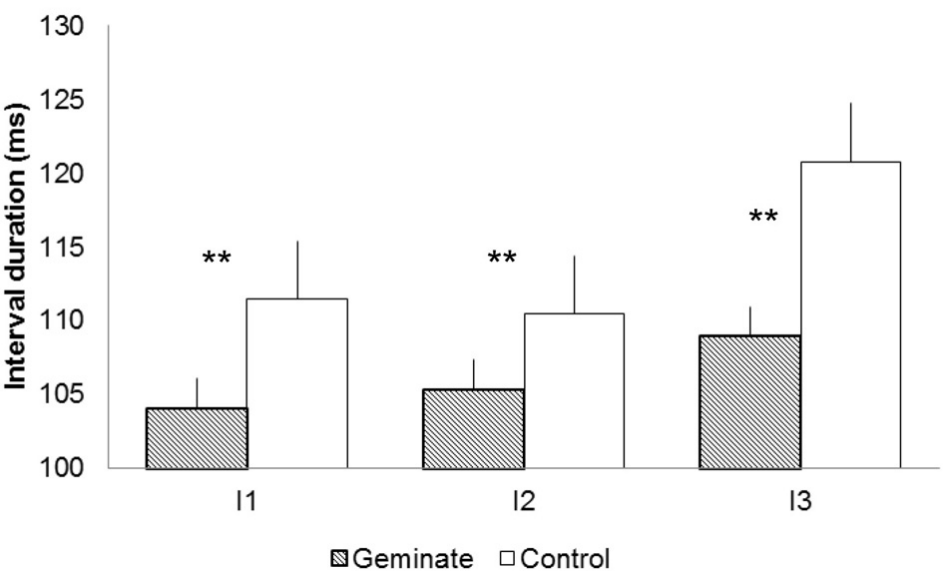
**Durations for intervals 1–3 [e.g., DISSIPATE/DISGRACE: Interval 1 (I1) = DI, Interval 2 (I2) = IS, Interval 3 (I3) = SS/SG]**. The ^*^ indicates a significant duration difference between Geminate and Control words at a specific location of the word; ^**^ indicates that the difference is significant for *F*1 and *F*2.

Figure 2 presents the mean durations for intervals 1–3 for Geminate and Control words. Geminate words yielded shorter interval durations than controls, *F*1_(1, 19)_ = 13.94, *p* < 0.001; *F*2_(1, 13)_ = 60.18, *p* < 0.001. Interval position yielded a significant effect, *F*1_(2, 38)_ = 6.15, *p* < 0.01; *F*2_(2, 26)_ = 6.09, *p* < 0.01. The interaction between the two factors was significant, *F*1_(2, 38)_ = 5.01, *p* < 0.01; *F*2_(2, 26)_ = 4.29, *p* < 0.05. Pairwise comparisons revealed that the interval durations for geminated words were shorter than controls at all positions: for I1, *F*1_(1, 19)_ = 6.28, *p* < 0.05; *F*2_(1, 13)_ = 13.76, *p* < 0.01; for I2, *F*1_(1, 19)_ = 4.96, *p* < 0.05; *F*2_(1, 13)_ = 7.02, *p* < 0.05; and for I3, *F*1_(1, 19)_ = 37.49, *p* < 0.001; *F*2_(1, 13)_ = 17.35, *p* < 0.001. The intervals remained stable from I2 to I3 (SS) in geminates (both F < 1) but increased significantly from I2 to I3 (SG) in controls, *F*1_(1, 19)_ = 17.35, *p* < 0.001; *F*2_(1, 13)_ = 0.39, *p* < 0.01 (Bonferroni corrected). Latency values correlated positively and significantly with interval duration for geminates at I3, *R* = 0.56, *p* < 0.01. For controls the correlations were significant for all interval positions: for I1, *R* = 0.67, *p* < 0.001; for I2, *R* = 0.56, *p* < 0.01; and for I3, *R* = 0.63, *p* < 0.01.

## Discussion

This research investigated whether central and peripheral processes interact during word writing. We examined whether the activation of letter doubling coding cascaded into motor production. The participants wrote English words on a digitizer. The words had a doublet embedded (e.g., DI*SS*IPATE) and were matched to words without a doublet that shared the initial letters (e.g., DI*SG*RACE). We measured latencies as an indicator of the time required for the activation of orthographic representations. We measured letter duration and the interval between the letters as indicators of peripheral processing. The results globally revealed that the production of Geminates differed from Controls. They suggest that letter doubling is a specific feature that modulates orthographic activation and motor production.

The results on latencies revealed that the activation of orthographic representations of Geminate words required less processing than controls. Once the participants started to write, we observed that the stroke durations for the four initial letters of Geminate words were systematically shorter than Control words. For the DI*SS*IPATE/DI*SG*RACE example, stroke durations for D (L1), I (L2) and S (L3), were shorter for Geminate than Control words. The interval data revealed the same pattern of results. Intervals 1–3 were systematically shorter for Geminates than Controls. Although bigrams DI and IS were identical in both conditions, the processing of the doublet accelerated movement production with respect to Controls. This indicates that the activation of the spelling processes before movement initiation (latency) cascaded into the peripheral processes that regulated movement production (letter and interval duration). This is evidence for an interaction between the two levels of processing.

The data for latencies, letter stroke durations, and interval durations converge. They all indicate that processing Geminate words is less time consuming than processing words not containing double letters. Furthermore, the latencies were positively correlated with duration values. This means that the central processes that were active before starting to write were still active during movement execution. Orthographic representations of Geminate words code the information on the presence of a doublet. This seems to facilitate spelling recall and decrease the processing load during movement production. Therefore, the central processes for spelling recovery are not completely finished before movement initiation. The writing system functions in a cascaded manner, as proposed by Delattre et al. (2006) and Roux et al. (2013).

The analysis also revealed that the difference between Geminates and Controls was significant at L4 and I3; i.e., the location at which the words differed. The durations remained stable from L3 to L4 and from intervals I2 to I3 in Geminates. Following the rationale presented in the models by Van Galen (1991) and Kandel et al. (2011), the writing system processed the doublet well before it occurred (before movement initiation and during the production of letters 1–3), so there is no reason for observing a duration difference between the two letters of the doublet (e.g., between the two S in DI*SS*IPATE). When they are produced, the processing only concerns the local aspects of letter production, which are the same for the two letters. So the presence of the doublet was anticipated and processed during movement preparation and later on throughout the production of the initial letters. There were no duration increases at I3 and L4 because - at the local level- producing the second S required the repetition of the motor program activated in L3. In controls, we observed increases in stroke duration from L3 to L4 and intervals I2 to I3 (e.g., from S to G in DI*SG*RACE). These increases suggest that in Controls L4 required much more processing than in Geminates. So in controls L4 was not programmed beforehand. Producing L4 required more than the regulation of local parameters for letter production.

The results are consistent with the idea that handwriting production functions in an anticipatory fashion, as posited in Van Galen's (1991) model. The writing system processed letter doubling before starting to write and throughout the production of the letters that preceded the doublet. They also support the idea put forward by Kandel et al.'s (2011) psycholinguistic model that orthographic representations are multi-dimensional. The writing system activates orthographic representations that code information on different kinds of letter clusters. This idea is in line with the neuropsychological data on dysgraphic patients (Tainturier and Caramazza, 1996). We observed that orthographic representations code the presence of a doublet in a word. This modulates the timing of motor production in such a way that the kinematics to produce a letter will depend on letter quantity rather than on letter identity. The results of the present study therefore have further implications than those of previous research on letter doubling. They provide evidence showing that the effects of letter clustering and processing are also observed in peripheral and late stages of writing.

A second implication of our study is that it provides information on *how* doublet processing affects word writing in its late stages. The on-line kinematic measures revealed that the processing of the doublet facilitated motor production. Finally, the third implication is that the measures on letter and interval duration can cast light on the *locus* of the processing. The results indicated that doublet processing starts well before starting to write and ends at interval 3 (e.g., after the first S in DI*SS*IPATE). This means that the activation of central spelling processes that were observed on latencies spread onto peripheral processing until the doublet was completely programmed. Lexical and sublexical variables cascade on the first or second letters of the word [except for irregular words with the irregularity at the end; (Roux et al., 2013)]. Doublet processing cascades until the gemination is actually produced.

### Conflict of interest statement

The first author, Sonia Kandel, is one of the editors of this Research Topic. The other authors declare that the research was conducted in the absence of any commercial or financial relationships that could be construed as a potential conflict of interest.

## Footnotes

^1^Orthographic regularity refers to the possibility of spelling a word correctly by using phono-graphemic conversion mappings. In French, for example, FEMME (/fam/, woman) is orthographically irregular because if we spell it by applying these rules we would incorrectly spell FAME. For FORME (/foRm/, shape) instead, there is no spelling ambiguity so we consider this word orthographically regular.

^2^Letter duration was normalized so that the durations of the preceding letters in both conditions could be compared. See the Data Analysis section for information on the procedure.

## Appendix

### Geminate and control words used in the experiment

Geminate words: ballot, carrier, collar, corridor, dissipate, dissolute, ferrous, manner, marrow, massive, passive, passion, pollute, pressure.

Control words: balcony, caring, colony, corporal, disgrace, distinct, fertile, manage, martial, master, pasting, pastor, polemic, prestige.

## References

[B1] Afonso O. Álvarez C. J. (2011). Phonological effects in handwriting production: Evidence from the implicit priming paradigm. J. Exp. Psychol. Learn. Mem. Cogn. 37, 1474–1483 10.1037/a0024515 21767055

[B2] Baayen R. H. Piepenbrock R. Gulikers L. (1995). The CELEX lexical database [CD-ROM]. Philadelphia: Linguistic Data Consortium, University of Pennsylvania

[B3] Bonin P. Peereman R. Fayol M. (2001). Do phonological codes constrain the selection of orthographic codes in written picture naming. J. Mem. Lang. 45, 688–720 10.1006/jmla.2000.2786

[B4] Cassar M. Treiman R. (1997). The beginnings of orthographic knowledge: Children's knowledge of double letters in words. J. Educ. Psychol. 89, 631–644 10.1037/0022-0663.89.4.631

[B5] Delattre M. Bonin P. Barry C. (2006). Written spelling to dictation: Sound-to-spelling regularity affects both writing latencies and durations. J. Exp. Psychol. Learn. Mem. Cogn. 32, 1330–1340 10.1037/0278-7393.32.6.1330 17087587

[B6] Danjon J. Pacton S. (2009). Children's learning about properties of double letters: the case of French, in Presented at the 16th European Society for Cognitive Psychology Conference (ESCOP), (Kraków)

[B7] Fayol M. Treiman R. Lété B. Pacton S. (2010). Learning to Spell from Reading: General Knowledge about Spelling Patterns can Distort Memory for Specific Words. St. Louis, MO: Psychonomic Society 10.1080/17470218.2013.846392 24224481

[B8] Glasspool D. W. Houghton G. (2005). Serial order and consonant-vowel structure in a graphemic output buffer model. Brain Lang. 94, 304–330 10.1016/j.bandl.2005.01.006 16098380

[B9] Guinet E. Kandel S. (2010). Ductus: A software package for the study of handwriting production. Behav. Res. Methods 42, 326–332 10.3758/BRM.42.1.326 20160312

[B10] Kandel S. Peereman R. Grosjacques G. Fayol M. (2011). For a psycholinguistic model of handwriting production: Testing the syllable-bigram controversy. J. Exp. Psychol. Learn. Mem. Cogn. 37, 1310–1322 10.1037/a0023094 21500939

[B11] Kandel S. Spinelli E. (2010). Processing complex graphemes in handwriting production. Mem. Cogn. 38, 762–770 10.3758/MC.38.6.762 20852239

[B12] Kandel S. Soler O. Valdois S. Gros C. (2006). Graphemes as motor units in the acquisition of writing skills. Read. Writ. 19, 313–337 10.1007/s11145-005-4321-5

[B13] Kawamoto A. H. Kello C. T. Jones R. Bame K. (1998). Initial phoneme versus whole-word criterion to initiate pronunciation: Evidence based on response latency and initial phoneme duration. J. Exp. Psychol. Learn. Mem. Cogn. 24, 862–885 10.1037/0278-7393.24.4.862

[B14] McCloskey M. Badecker W. Goodman-Schulman R. A. Aliminosa D. (1994). The structure of graphemic representations in spelling: Evidence from a case of acquired dysgraphia. Cogn. Neuropsychol. 11, 341–392 10.1080/02643299408251979

[B15] Miceli G. Benvengnú B. Capasso R. Caramazza A. (1995). Selective deficit in processing double letters. Cortex 31, 161–171 10.1016/S0010-9452(13)80114-1 7781313

[B16] Pacton S. Borchardt G. Treiman R. Lété B. Fayol M. (in review). Learning to spell from reading: general knowledge about spelling patterns influences memory for specific words. 10.1080/17470218.2013.846392 24224481

[B17] Pacton S. Perruchet P. Fayol M. Cleeremans A. (2001). Implicit learning out of the lab: The case of orthographic regularities. J. Exp. Psychol. Gen. 130, 401–426 10.1037/0096-3445.130.3.401 11561917

[B18] Rabiner L. R. Gold B. (1975). Theory and application of digital signal processing. Upper Saddle River, NJ: Prentice-Hall

[B19] Rapp B. Epstein C. Tainturier M.-J. (2002). The integration of information across lexical and sublexical processes in spelling. Cogn. Neuropsychol. 19, 1–29 10.1080/0264329014300060 20957529

[B20] Rastle K. Harrington J. Palethorpe S. Coltheart M. (2000). Reading aloud begins when the computation of phonology is complete. J. Exp. Psychol. Hum. Percept. Perform. 26, 1178–1191 10.1037/0096-1523.26.3.1178 10884016

[B21] Roux J.-S. McKeeff T. J. Grosjacques G. Afonso O. Kandel S. (2013). The interaction between central and peripheral processes in handwriting production. Cognition 2, 235–241 10.1016/j.cognition.2012.12.009 23454797

[B22] Sternberg S. Knoll R. L. Monsell S. Wright C. E. (1983). Control of Rapid Action Sequences in Speech and Typing. Murray Hill, NJ: ATandT Bell Laboratories

[B23] Sternberg S. Knoll R. L. Turock D. L. (1990). Hierarchical control in the execution of action sequences: Test of two invariance properties, in Attention and Performance XIII: Motor Representation and Control, ed Jeannerod M. (Hillsdale, NJ: Erlbaum), 3–55

[B24] Teulings H. L. Thomassen A. J. W. M. Van Galen G. P. (1983). Preparation of partly precued handwriting movements: The size of movement units in handwriting. Acta Psychol. 54, 165–177 10.1016/0001-6918(83)90031-8

[B25] Qu A. Damian M. F. Zhang Q. Zhu X. (2011). Phonology contributes to writing: Evidence from written word production in a nonalphabetic script. Psychol. Sci. 22, 1107–1112 10.1177/0956797611417001 21775652

[B26] Spinelli E. Kandel S. Guerassimovitch H. Ferrand L. (2012). Graphemic cohesion effect in reading and writing complex graphemes. Lang. Cogn. Process. 27, 770–791 10.1080/01690965.2011.586534

[B27] Tainturier M. J. Caramazza A. (1996). The status of double letters in graphemic representations. J. Mem. Lang. 36, 53–73 10.1006/jmla.1996.0003

[B28] Van Galen G. P. (1991). Handwriting: Issues for a psychomotor theory. Hum. Mov. Sci. 10, 165–191 10.1016/0167-9457(91)90003-G

[B29] Van Galen G. P. Smyth M. M. Meulenbroek R. G. J. Hylkema H. (1989). The role of short-term memory and the motor buffer in handwriting under visual and non-visual guidance, in Computer Recognition and Human Production of Handwriting, eds Plamondon R. Suen C. Y. Simner M. L. (Singapore: World Scientific), 253–271

[B30] Venneri A. Cubelli R. Caffara P. (1994). Perseverative dysgraphia: A selective disorder in writing double letters. Neuropsychologia 32, 923–931 10.1016/0028-3932(94)90043-4 7969867

[B32] Weingarten R. (2005). Subsyllabic units in written word production. Writ. Lang. Lit. 8, 43–61 10.1075/wll.8.1.03wei

[B33] Weingarten R. Nottbusch G. Will U. (2004). Morphemes, syllables, and graphemes in written word production, in Multidisciplinary Approaches to Language Production, eds Pechmann T. Habel C. (Berlin: Mouton de Gruyter), 529–572

[B34] Wright D. M. Ehri L. C. (2007). Beginners remember orthography when they learn to read words: The case of double letters. Appl. Psychol. 28, 115–133 10.1017/S0142716406070068

[B35] Yarkoni T. Balota D. Yap M. (2008). Moving beyond Coltheart's N: A new measure of orthographic similarity. Psychon. Bull. Rev. 15, 971–979 10.3758/PBR.15.5.971 18926991

[B36] Zhang Q. Damian M. F. (2010). Impact of phonology on the generation of handwritten responses: Evdience from picture-word interference tasks. Mem. Cogn. 38, 519–528 10.3758/MC.38.4.519 20516232

